# Real-world changes in lipid-lowering therapy use and LDL-C goal attainment in high and very high cardiovascular risk patients in the UK: a secondary analysis of the European SANTORINI study 1-year follow-up

**DOI:** 10.1136/bmjopen-2025-114031

**Published:** 2026-04-24

**Authors:** Derek Connolly, Ahmet Fuat, Terry McCormack, Damien Mcnally, Jonathan Garstang, John Ryan, Amelia Reed, Daniel Robinson, Alberico L Catapano, Kausik K Ray

**Affiliations:** 1 Midland Metropolitan University Hospital, Birmingham City Hospital, Institute of Cardiovascular Sciences, University of Birmingham, and Aston Medical School, Sandwell and West Birmingham Hospitals NHS Trust, Birmingham, UK; 2 Durham University, Durham, UK; 3 Institute of Clinical and Applied Health Research, University of Hull, Hull York Medical School, Hull, UK; 4 Ormeau Clinical Trials, Ormeau Health Centre, Belfast, UK; 5 Knowle House Surgery, Plymouth, UK; 6 The Alverton Practice, Truro, UK; 7 Medical Department, Daiichi Sankyo UK Ltd, Uxbridge, UK; 8 Department of Pharmacological and Biomolecular Sciences, University of Milan, Milan, Italy; 9 Multimedica IRCCS Via Milanese, Milan, Italy; 10 Imperial Centre for Cardiovascular Disease Prevention, Imperial College London, London, UK

**Keywords:** Cardiovascular Disease, Lipid disorders, Treatment Outcome, REGISTRIES

## Abstract

**Abstract:**

**Objectives:**

This real-world study investigated the changes of lipid lowering therapy (LLT) usage in patients with high or very high cardiovascular (CV) risk in the UK and the group of all other European countries in the SANTORINI study up to 1 year from baseline and the impact this treatment had on the attainment of low-density lipoprotein cholesterol (LDL-C) risk-adjusted goals set by the National Institute for Health and Care Excellence (NICE) and those in the 2019 European Society of Cardiology (ESC)/European Atherosclerosis Society (EAS) dyslipidaemia guidelines.

**Design:**

Secondary analysis of the SANTORINI dataset (an international, prospective, observational, non-interventional study (NCT04271280)).

**Setting:**

Primary and secondary care centres in the UK and the group of other European countries (Austria, Belgium, Denmark, Finland, France, Germany, Ireland, Italy, the Netherlands, Portugal, Spain, Sweden and Switzerland).

**Participants:**

663 UK patients with high and very high CV risk were included in this analysis and 8502 from the group of other European countries. Of these, 380 UK patients and 6830 from the group of other European countries had LDL-C information available at baseline and 1-year follow-up.

**Primary outcome measures:**

The primary objectives were to describe patients’ lipid management, LDL-C levels at 1-year follow-up and their attainment of 2023 NICE (≤2.0 mmol/L) and 2019 ESC/EAS LDL-C 2019 guideline-recommended LDL-C goals (<1.4 mmol/L for very high-risk patients and <1.8 mmol/L for high-risk patients) within this time frame.

**Results:**

Over the course of 1-year follow-up, the overall proportion of UK patients on no LLT reduced from 20.4% at baseline to 7.1%, similar to that observed in the group of other European countries (baseline–20.9%, 1 year–3.0%). The proportion of UK patients receiving LLT monotherapy increased from 74.8% at baseline to 84.9%, higher at both time points than that observed for the group of other European countries (baseline: 52.0%, 1 year: 55.0%). The use of any combination therapy increased slightly from baseline to 1 year in the UK overall cohort (4.9% vs 7.1%) and overall in the group of all other European countries, the cohort increased from baseline (27.1%) to 1 year (40.2%). Overall, mean (SD) LDL-C levels in the UK were 2.5 (1.2) mmol/L at baseline and 2.1 (1.0) mmol/L at 1 year and for the group of other European countries were 2.4 (1.2) mmol/L at baseline and 2.0 (0.9) mmol/L at 1 year. The overall proportions of UK patients achieving the UK NICE treatment goal and ESC/EAS 2019 guidelines at baseline versus 1-year follow-up were 40.3% vs 52.6% and 22.9% vs 32.9%, respectively; 21.1% and 30.9% of patients in the group of other European countries achieved the ESC/EAS 2019 guidelines at baseline and 1-year follow-up, respectively.

**Conclusions:**

In this UK-focused analysis of the SANTORINI study, use of LLT increased modestly over 1 year, accompanied by a reduction in average LDL-C levels. However, mean LDL-C remained above the NICE goal, and attainment of both NICE and ESC/EAS LDL-C thresholds remained suboptimal. The findings highlight continued opportunities to optimise lipid management in UK clinical practice, including the potential for broader use of combination therapies.

**STRENGTHS AND LIMITATIONS OF THIS STUDY**

- This study uses prospectively collected data to investigate the usage of lipid lowering therapies and attainment of low-density lipoprotein cholesterol (LDL-C) goals in UK patients with high or very high risk of cardiovascular disease reflecting how LDL-C levels are managed in UK clinical practice.
- This is one of the largest European observational studies performed since the 2019 lipid guidelines were published.
- The SANTORINI study was conducted in 14 countries across Europe, enabling comparison of the clinical management of LDL-C levels across countries.
- The study was descriptive, and no analysis was performed to control for possible confounders.
- Prospectively followed patients may be subject to Hawthorne bias.

## Introduction

 Cardiovascular disease (CVD) remains a leading cause of death in the UK, with over 7 million people living with heart and circulatory diseases.^1^ High levels of low-density lipoprotein cholesterol (LDL-C) are a significant contributor to CV risk, and managing these levels is crucial for preventing atherosclerotic CVD (ASCVD).^2^ Lipid-lowering therapies (LLTs), particularly those targeting LDL-C, are fundamental in reducing CV risk for patients. These therapies, including statins, ezetimibe, bempedoic acid and proprotein convertase subtilisin/kexin type 9 (PCSK9) targeted therapies, work principally by lowering LDL-C levels.

Clinical guidelines emphasise the importance of achieving and maintaining low LDL-C levels, with LDL-C goals set according to patients’ CVD risk status, to minimise the risk of fatal and non-fatal CV events such as myocardial infarction, stroke and coronary artery disease.^3 4^ Combining these pharmacological treatments with lifestyle modifications, such as a healthy diet and regular exercise, further supports their effectiveness in reducing CV risk. In the UK, National Institute for Health and Care Excellence (NICE) 2023 guidelines recommend an LDL-C goal of ≤2.0 mmol/L for the secondary prevention of CVD (with and without type 1 or 2 diabetes),^4^ while the 2019 European Society of Cardiology (ESC)/European Atherosclerosis Society (EAS) dyslipidaemia guideline recommendations are more stringent and stratify for patient CVD risk status with the recommendations set at <1.8 mmol/L and <1.4 mmol/L for patients at high risk and very high risk of CVD, respectively.^3^ Despite these guidelines, achieving LDL-C goals remains challenging, with many patients not reaching the recommended levels.^5^,^7^

Studies such as the ASPIRE-3-PREVENT survey (using data from 5 English National Health Service regions) have highlighted gaps in the implementation of CV prevention strategies in primary care.^5^ This survey found that a significant proportion of patients did not achieve LDL-C goals and many were not receiving adequate LLT. Continuous monitoring and adjustment of treatment plans are essential to improve LDL-C control and reduce the risk of CV events.^8^ While progress has been made in reducing CVD mortality in the UK, there is still a need for better implementation of guidelines and more effective management of LDL-C levels to prevent ASCVD and improve CV health outcomes.^5 8^

In this UK-focused analysis of the prospective follow-up of the SANTORINI cohort (completed prior to the introduction of the 2023 NICE guidelines),^4^ we examine changes in LLT usage patterns and their impact on LDL-C goal attainment after 1 year according to both 2023 NICE and 2019 ESC/EAS guideline goals.

As part of the broader SANTORINI study programme, several related publications have previously characterised baseline treatment gaps across Europe, including the finding that approximately 80% of high-risk and very high-risk patients failed to achieve guideline-recommended LDL-C goals at enrolment.^9^ Further secondary analyses from the same cohort have also explored variations in LDL-C management, such as the age-stratified evaluation of LLT use and goal attainment at 1-year follow-up^10^ and country-level analyses (eg, Ireland and France demonstrate persistent challenges in achieving LDL-C targets despite treatment intensification).^11 12^ These related analyses provide context for the current UK-focused secondary analysis, reinforcing the ongoing need to understand treatment patterns and address LDL-C management gaps across diverse European healthcare settings.

## Methods

### Study design and objectives

The current study presents the data collected from UK participants within the SANTORINI study. The SANTORINI study (NCT04271280) was a prospective, observational study in patients with high and very high CV risk across 14 European countries. Patients were recruited from primary and secondary care sites between 17 March 2020 and 11 February 2021 and followed up to 1 year post recruitment (~12±2 months). The study database was locked on 31 May 2022. The rationale and methods used have been described previously,^13^ but in summary, patients were eligible to participate if they were ≥18 years with high or very high CV risk (CV risk was assigned by the study investigators at study enrolment, with the basis for the risk category documented), if they required LLT, had an anticipated life expectancy over 1 year from enrolment, were not participating in any simultaneous interventional studies and provided written informed consent to participate (see online supplemental figure 1 for patient flow diagram).

Based on the 2019 ESC/EAS guideline criteria, very high-risk patients were those documented with ASCVD, diabetes mellitus, type 1 diabetes mellitus with target organ damage or additional major risk factors such as smoking, marked hypercholesterolaemia or hypertension, moderate or severe chronic kidney disease (estimated glomerular filtration rate (eGFR)<30 mL/min), or calculated SCORE 10-year risk for fatal CVD ≥10% while high-risk patients are those with a significantly elevated single risk factor such as total cholesterol (TC) >8 mmol/L (>310 mg/dL), familial hypercholesterolaemia (FH) and elevated blood pressure, patients with diabetes mellitus with or without target organ damage or for more than 10 years, moderate chronic kidney disease (eGFR 30–59 mL/min), or calculated SCORE 10-year risk for fatal CVD ≥5 and <10%.

The objective of the current study was to use the UK SANTORINI dataset and describe the current patterns of LLT usage in the UK up to 1 year from baseline, and the impact this treatment had on the attainment of LDL-C risk-adjusted goals set by NICE and those in the 2019 ESC/EAS dyslipidaemia guidelines.

### Study outcomes and data collection

The main outcomes assessed were to describe LLT treatment in UK patients during the 1 -year follow-up, and how this impacted the attainment of LDL-C goals set by NICE and/or 2019 ESC/EAS dyslipidaemia guidelines with participants presented overall and stratified by CV risk level. The 2019 ESC/EAS guidelines stipulate that the thresholds are <1.4 mmol/L for patients at very high risk and <1.8 mmol/L for patients at high risk,^3^ while the NICE guidelines (introduced in 2023 for the secondary prevention of CVD) stipulate a threshold of ≤2.0 mmol/L for patients with and without type 1 or 2 diabetes.^4^ Data collected at baseline included patients’ baseline characteristics and medical history, current LLT and co-medications as well as LLT from the prior year for those who were previously diagnosed and treated. At the 12-month follow-up visit, available data were collected on patient management between baseline and data collection point including information on efficacy (LDL-C plasma levels), treatment management (prescribed LLTs, treatment escalation/de-escalation) and outcomes.

Reporting of this observational study adheres to the Strengthening the Reporting of Observational Studies in Epidemiology guidelines.

### Statistical analyses

This analysis used the full analysis set (FAS) from the UK cohort of the SANTORINI study and uses the remaining European data to provide further context for this UK data. Data were analysed descriptively, and no comparative analyses were performed. Distributions and descriptive statistics of central tendency (medians and arithmetic or geometric means) and dispersion (SD, IQR, range) were presented for quantitative variables. Categorical variables were described with frequencies and percentages. No imputation was performed for missing data. Analyses are presented as follows for both the UK FAS and for the group of other European countries: overall, for those with LDL-C data available (baseline and 1-year follow-up), and stratified according to investigator-assessed risk classification at baseline (high or very high risk of CV). All statistical analyses were performed using SAS V.9.4.

### Patient and public involvement

Patients and/or the public were not involved in the design, or conduct, or reporting, or dissemination plans of this research.

## Results

### Patient demographics and clinical characteristics

A total of 663 UK patients were documented and screened for inclusion in the SANTORINI study of whom 634 had 1-year follow-up data and were included in the study (see online supplemental figure 1). Of these 634 UK patients, 380 had LDL-C data available at both baseline and 1-year follow-up, 346 patients were classed as high risk and 287 as very high CV risk, respectively, by investigators at enrolment. Of the 8502 patients that were included in the SANTORINI study from across the other 13 European countries, 6830 had LDL-C data available at baseline and 1-year follow-up, with 2280/6217 patients classed as high risk/very high risk, respectively, by investigators at enrolment.

Overall, 67.2% of UK patients were male with a mean (SD) age of 67.0 (9.0) years, mean (SD) BMI of 30.3 (6.1) kg/m^2^ and a mean LDL-C of 2.4 mmol/L (94.2 mg/dL). Baseline demographics and clinical characteristics were similar in both the overall and LDL-C UK data sets (online supplemental table 1). A higher proportion of patients from the group of other European countries was male (73.2%), they were younger on average (mean age, 65.3 years) with a marginally lower mean BMI (28.2 kg/m^2^) and similar mean LDL-C (2.4 mmol/L). Baseline demographics and clinical characteristics were broadly similar across UK subgroups, though a higher proportion of patients in the very high-risk group were male (77.0% vs 59.0%), had ASCVD (58.5% vs 30.6%) and had heterozygous FH (8.0% vs 5.2%) when compared numerically with those in the high-risk group (online supplemental table 1). Mean LDL-C was also numerically lower in the very high-risk group (2.3 mmol/L) than in the high-risk group (2.6 mmol/L) for the UK and the group of other European countries (high-risk group–2.7 mmol/L, very high-risk group–2.3 mmol/L).

### LLT use at baseline and end of follow-up

**Figure 1:**
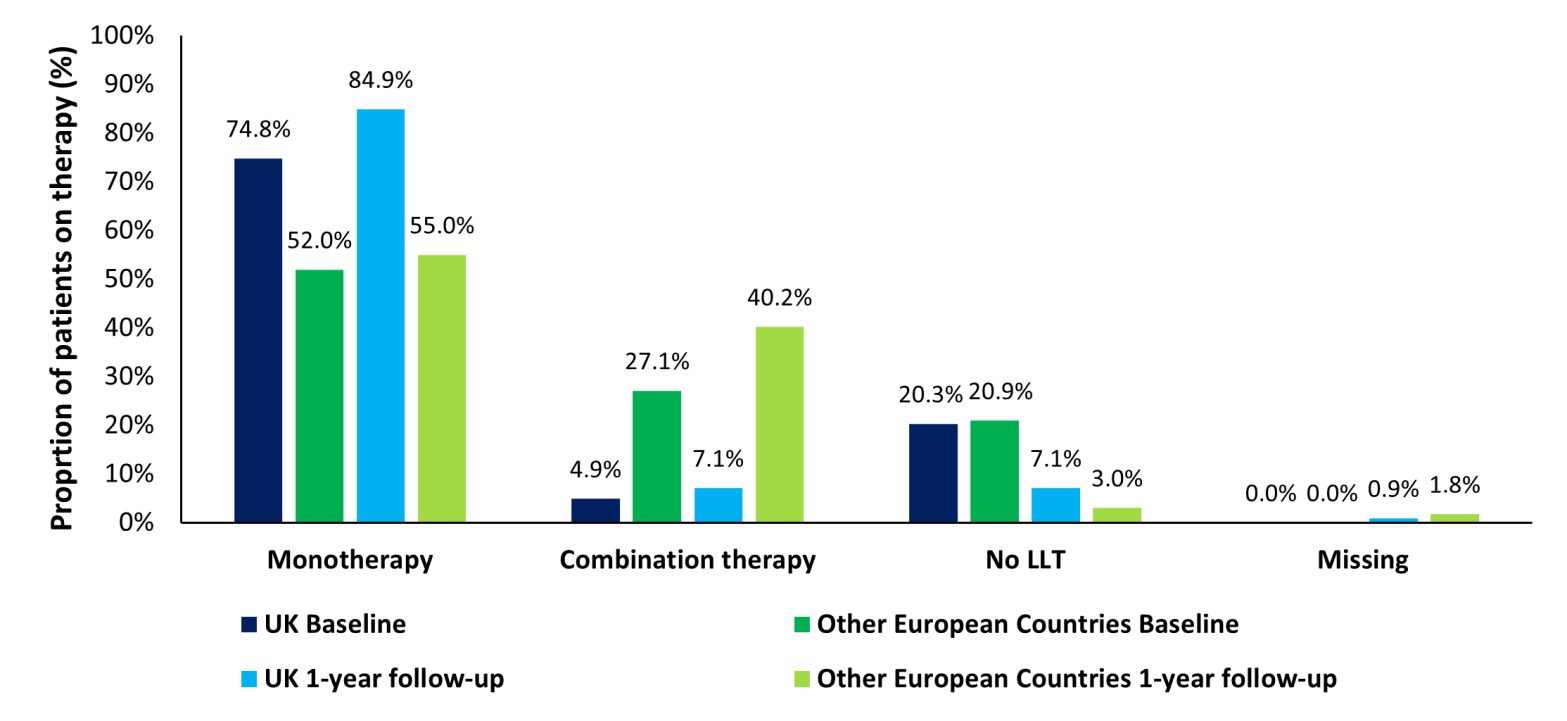
Overall usage in the UK cohort (n=634) and other European countries (n=8502) of lipid lowering monotherapy and combination therapy at baseline and 1-year follow-up. LLT, lipid-lowering therapy*.*

Use of LLTs at baseline and 1-year follow-up in the UK and in the group of other European countries is summarised in figure 1 and online supplemental tables 2 and 3. Over the course of 1-year follow-up, the proportion of UK patients (overall) on no LLT reduced from 20.4% at baseline to 7.1%, similar to that observed in the group of other European countries (figure 1). Stratified by high-risk and very high-risk status, the proportions of UK patients receiving no LLT fell from 23.4% to 9.5% and from 16.7% to 4.2%, respectively, with proportions in either risk group at 1 year still higher than that observed for the group of other European countries (high-risk–5.2%, very high-risk–2.2%; online supplemental tables 2 and 3). Over the course of 1-year follow-up, the proportion of UK patients (overall) receiving LLT monotherapy increased from 74.8% at baseline to 84.9%, higher at both time points than that observed for the group of other European countries (baseline–52.0%, 1 year–55.0%; figure 1). Stratified by high-risk and very high-risk status, the proportions of UK patients receiving LLT monotherapy increased to a similar degree from 74.3% to 84.7% and from 75.3% to 85.0%, respectively (online supplemental tables 2 and 3).

Overall, the UK prescribing patterns of statin monotherapy at baseline and 1-year follow-up were: 1.1% vs 0.6%, 43.9% vs 49.1% and 25.1% vs 30.9% for low, moderate and high-intensity statin use, respectively (online supplemental table 2). When stratified by risk status, the prescribing patterns in high-risk (very high-risk) patients at baseline and 1-year follow-up were: 0.6% vs 0.3% (1.7% vs 1.1%), 49.7% vs 56.1% (36.6% vs 40.4%), and 19.9% vs 24.9% (31.4% vs. 38.3%) for low, moderate and high-intensity statin use, respectively (online supplemental table 2). Respectively, in the group of other European countries, the prescribing patterns of statin monotherapy at baseline and 1-year follow-up were: 1.5% vs 1.3%, 24.2% vs 22.9% and 21.2% vs 25.4% for low, moderate and high-intensity statin use, respectively. Prescribing patterns for the group of other European countries when stratified by risk status, in high-risk (very high-risk) patients at baseline and 1-year follow-up were: 0.6% vs 0.3% (1.7% vs. 1.1%), 49.7% vs 56.1% (36.6% vs. 40.4%) and 19.9% vs 24.9% (31.4% vs. 38.3%) for low, moderate and high-intensity statin use, respectively (online supplemental table 3). Changes in the use of other oral LLT monotherapies over 1 year such as ezetimibe, bempedoic acid and PCSK9 targeted therapies were small (<2%) overall and across risk status groups both in the UK and for the group of other European countries (online supplemental tables 2and 3).

Use of any combination therapy increased slightly from baseline to 1 year in the UK overall cohort (4.9% vs 7.1%) and in both risk status groups (high-risk–2.3% vs 5.2%; very high-risk–8.0% vs 9.4%) (online supplemental table 2, online supplemental figure 2). The proportional use of any combination therapy overall, in the group of all other European countries cohort, increased from baseline (27.1%) to 1 year (40.2%) and in both risk status groups (high-risk–21.6% vs 30.2%; very high-risk–29.1% vs 43.9%) (online supplemental table 3). Overall, this increase in combination therapy was equal for the use of PCSK9 combinations and statin+ezetimibe combinations which increased from baseline to 1 year from 1.4% to 2.4% and from 1.4% to 2.4%, respectively. Patients in the high-risk group saw larger increases in usage for both combinations at 1 year (PCSK9 combination—0.9% vs 2.0%; statin+ezetimibe combinations—1.2% vs 2.3%) than patients in the very high-risk group (PCSK9 combination—2.1% vs 2.8%; statin+ezetimibe combinations—4.2% vs 4.9%) (online supplemental table 2). For patients in the group of other European countries, statin+ezetimibe combinations were the most prevalent combination therapy (18.2% at baseline, 28.1% at 1 year; online supplemental table 3). This was true for both risk status groups.

Overall, 75.4% of UK patients had no reported change in their treatment intensity classification, 21.1% had had an escalation in treatment and 2.5% of patients had had a de-escalation (online supplemental table 4). For UK patients at high risk (very high risk), 72.8% (78.4%) of patients had no reported change in their treatment intensity classification, 22.8% (19.2%) had had an escalation in treatment and 3.8% (1.1%) of patients had had a de-escalation. Overall, 65.9% of patients from the group of other European countries had no reported change in their treatment intensity classification, 29.9% had an escalation in treatment and 2.5% of patients had had a de-escalation (online supplemental table 4). For patients from the group of other European countries at high risk (very high risk), 70.3% (64.3%) of patients had no reported change in their treatment intensity classification, 26.5% (31.1%) had had an escalation in treatment, and 1.9% (2.7%) of patients had had a de-escalation.

In the UK FAS (n = 634), category counts moved from baseline to 1 year as follows: ‘no LLT’ 129 (20.4%) to 45 (7.1%), monotherapy 474 (74.8%) to 538 (84.9%) and combination therapy 31 (4.9%) to 45 (7.1%); 6 participants were missing at follow-up (online supplemental table 2). This net pattern indicates that the increase in monotherapy was driven predominantly by initiation among previously untreated patients, with only a minor contribution from category changes. In keeping with the treatment-intensification summary (75.4% no change; 21.1% escalation; 2.5% de-escalation), most escalation occurred within monotherapy intensity brackets (online supplemental table 4) and therefore did not alter treatment category, while de-escalation from combination to monotherapy was uncommon.

Patterns of treatment intensification also contribute to understanding the LDL-C changes observed at 1 year. As shown in online supplemental table 4, most UK patients had no change in their treatment intensity (478/634; 75.4%), while 134 (21.1%) experienced escalation and 16 (2.5%) underwent de-escalation. These escalation events predominantly increased the intensity of existing monotherapy regimens, as reflected by the small rise in combination therapy use (+14 patients). When considered alongside the substantial reduction in patients on no LLT (129 to 45), these findings indicate that the LDL-C reductions observed in the UK cohort were driven mainly by initiation of therapy in previously untreated individuals and intensification within monotherapy, rather than by widespread adoption of combination therapy.

### Risk-based LDL cholesterol change and goal attainment at baseline and follow-up

Table 1 summarises the changes in LDL-C at baseline and 1-year follow-up in the overall UK LDL-C dataset and stratified by CV risk at baseline. Overall, mean (SD) LDL-C levels in the UK were 2.5 (1.2) mmol/L at baseline and 2.1 (1.0) mmol/L at 1 year. In the high-risk and very high-risk groups, mean (SD) LDL-C levels were 2.6 (1.2) mmol/L and 2.4 (1.2) mmol/L at baseline, and 2.2 (1.0) mmol/L and 2.1 (1.0) mmol/L at 1 year, respectively. Mean (SD) LDL-C levels for the group of other European countries were 2.4 (1.2) mmol/L at baseline and 2.0 (0.9) mmol/L at 1 year. In the high-risk and very high-risk groups, mean (SD) LDL-C levels were 2.8 (1.3) mmol/L and 2.3 (1.2) mmol/L at baseline and 2.3 (1.0) mmol/L and 1.9 (0.9) mmol/L at 1 year, respectively, for the group of other European countries.

**Table 1:** Baseline and 1-year follow-up LDL-C and proportion of patients achieving ESC/EAS/NICE guideline recommended LDL-C goal at baseline and 1-year follow-up by baseline investigator risk (LDL-C dataset)

		UK						Other European countries					
		**Overall**		**High risk**		**Very high risk**		**Overall**		**High risk**		**Very high risk**	
		**Baseline**	**1-year follow- up**	**Baseline**	**1-year follow- up**	**Baseline**	**1-year follow- up**	**Baseline**	**1-year follow- up**	**Baseline**	**1-year follow- up**	**Baseline**	**1-year follow- up**
Number of patients (N)		380		199		181		6830		1834		4992	
LDL-C (mmol/L)*	n (%)	380 (100)	380 (100)	199 (100)	199 (100)	181 (100)	181 (100)	6830 (100)	6830 (100)	1834 (100)	1834 (100)	4992 (100)	4992 (100)
	Mean (SD)	2.5 (1.2)	2.1 (1.0)	2.6 (1.2)	2.2 (1.0)	2.4 (1.2)	2.1 (1.0)	2.4 (1.2)	2.0 (0.9)	2.8 (1.3)	2.3 (1.0)	2.3 (1.2)	1.9 (0.9)
Goal attainment													
EAS/ESC†‡	n (%)	87 (22.9)	125 (32.9)	59 (29.7)	82 (41.2)	28 (15.5)	43 (23.8)	1442 (21.1)	2107 (30.9)	551 (24.2)	–	1171 (18.8)	–
	(95% CI)	(18.8% to 27.5%)	(28.2% to 37.9%)	(23.4% to 36.5%)	(34.3% to 48.4%)	(10.5% to 21.6%)	(17.8% to 30.6%)	(20.2 to 22.1)	(29.8 to 32.0)	–	–	–	–
UK NICE‡	n (%)	153 (40.3)	200 (52.6)	72 (36.2)	99 (49.8)	81 (44.8)	101 (55.8)	–	–	–	–	–	–
	(95% CI)	(35.3% to 45.4%)	(47.5% to 57.8%)	(29.5% to 43.3%)	(42.6% to 56.9%)	(37.4% to 52.3%)	(48.3% to 63.2%)	–	–	–	–	–	–
% difference‡§	n (%)	66 (17.4)	75 (19.7)	13 (6.5)	17 (8.5)	53 (29.3)	58 (32.0)	–	–	–	–	–	–
	(95% CI)	(13.7% to 21.6%)	(15.9% to 24.1%)	(3.5% to 10.9%)	(5.1% to 13.3%)	(22.8% to 36.5%)	(25.3% to 39.4%)	–	–	–	–	–	–

* The denominator for n is the number of subjects in the population and in corresponding group.

† Follow-up LDL <1.4 mmol/L (very high risk patient at follow-up) or <1.8 mmol/L (high risk patient at follow-up) NICE goal attainment=(LDL-C ≤2.0 mmol/L).

‡ 95% CIs according to Clopper-Pearson.

§ Percentage difference: High-risk: (Patients with LDL-C <2.0 mmol/L and ≥1.8 mmol/L/N (high risk))×100. Very high-risk patients: (Patients with LDL-C <2.0 mmol/L and LDL-C ≥1.4 mmol/L/N (very high-risk))×100. Combined: (Patients with LDL-C <2.0 mmol/L and ≥1.8 mmol/L/N (high risk)+patients with LDL-C <2.0 mmol/L and ≥1.4 mmol/L/N (very high-risk))/overall N×100.

ESC/EAS, European Society of Cardiology/European Atherosclerosis Society; LDL-C, low-density lipoprotein cholesterol; NICE, National Institute for Health and Care Excellence.

UK patients not receiving LLT at baseline (n=90) had mean (SD) LDL-C of 3.5 (1.1) mmol/L at baseline and 2.2 (1.1) mmol/L at 1-year follow-up. Of these 90 patients, those receiving monotherapy at 1-year follow-up (n=72) had mean LDL-C (SD) of 3.2 (1.0) mmol/L at baseline and 1.8 (0.7) mmol/L at follow-up, while those receiving combination therapy at 1-year follow-up (n=2) had mean LDL-C (SD) of 4.3 (0.9) mmol/L at baseline and 1.4 (0.9) mmol/L at follow-up. The mean (SD) LDL-C of patients from the group of other European countries not receiving LLT at baseline (n=1467) at baseline and at 1-year follow-up was 3.5 (1.3) mmol/L and 2.2 (1.1) mmol/L, respectively. UK patients receiving LLT at baseline (n=290) had mean (SD) LDL-C of 2.2 (1.1) mmol/L at baseline and 2.1 (1.0) mmol/L at 1 years follow-up. Of these 290 patients, those receiving monotherapy at 1-year follow-up (n=258) had mean LDL-C (SD) of 2.1 (1.0) mmol/L at baseline and 2.1 (0.9) mmol/L at follow-up, while those receiving combination therapy at 1-year follow-up (n=25) had mean LDL-C (SD) of 2.8 (1.4) mmol/L at baseline and 2.1 (1.4) mmol/L at follow-up. Patients from the group of other European countries receiving LLT at baseline (n=5363) had mean (SD) LDL-C of 2.1 (1.0) mmol/L at baseline and 1.9 (0.9) mmol/L at 1-year follow-up.

**Figure 2:**
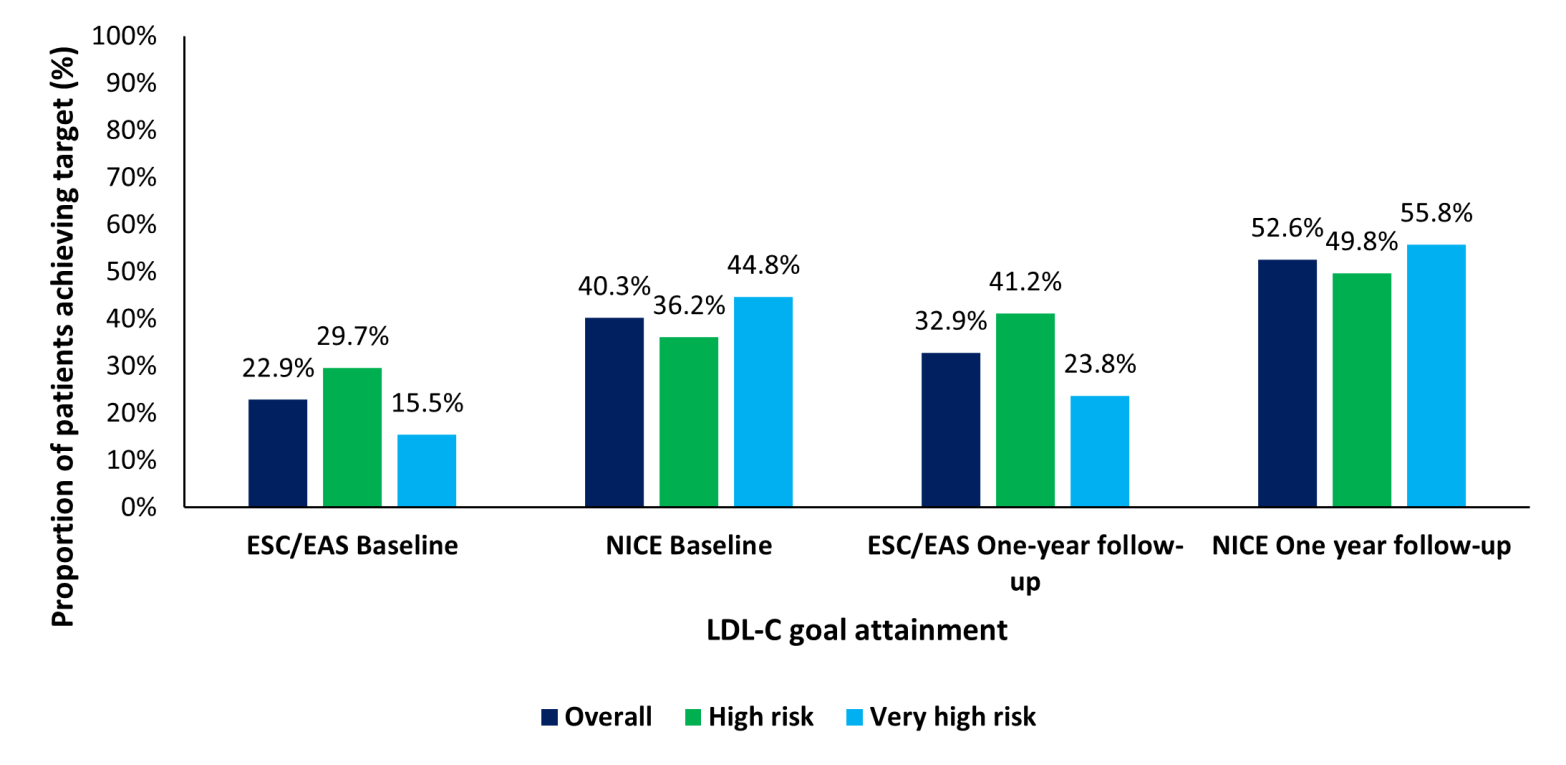
LDL-C NICE goal attainment at baseline and 1-year follow-up (UK cohort LDL-C data set, n=380). ESC/EAS, European Society of Cardiology/European Atherosclerosis Society; LDL-C, low-density lipoprotein cholesterol; NICE, National Institute for Health and Care Excellence.

Regarding treatment goal attainment, the overall proportions of patients achieving the UK NICE treatment goal (≤2.0 mmol/L) and ESC/EAS guidelines (<1.4 mmol/L for very high-risk patients and <1.8 mmol/L for high-risk patients) at baseline versus 1-year follow-up were 40.3% vs 52.6% and 22.9% vs 32.9%, respectively (table 1, figure 2); 21.1% and 30.9% of patients in the group of other European countries achieved the ESC/EAS guidelines at baseline and 1-year follow-up, respectively. The percentage difference in all patients achieving NICE and ESC/EAS goal LDL-C was 17.4% and 19.7% at baseline and 1-year, respectively. For patients in the high-risk group, the proportions of patients achieving the UK NICE treatment goal and ESC/EAS guidelines at baseline versus 1-year follow-up were 36.2% vs 49.8% and 29.7% vs 41.2%, respectively. The percentage difference in high-risk patients achieving NICE and ESC/EAS goal LDL-C was 6.5% and 8.5% at baseline and 1 year, respectively. For patients in the very high-risk group, the proportions of patients achieving the UK NICE treatment goal and ESC/EAS guidelines at baseline versus 1-year follow-up were 44.8% vs 55.8% and 15.5% vs 23.8%, respectively. The percentage difference in very high-risk patients achieving NICE and ESC/EAS goal LDL-C was 29.3% and 32.0% at baseline and 1 year, respectively.

## Discussion

These UK-focused findings, taken from the larger European SANTORINI study, provide key insights into the patient characteristics, clinical management, and achievement of treatment target goals for UK patients with dyslipidaemia between 2020 and 2022. We observed an average improvement in LDL-C levels of 0.5 mmol/L, which was driven predominantly by improvements observed in the high-risk subgroup of patients who displayed an improvement of 0.4 mmol/L, in contrast to the improvement of 0.3 mmol/L observed in the very high-risk group. Subsequently, this improvement in LDL-C translated into improved attainment of both NICE UK and ESC/EAS guideline-recommended LDL-C goals. Overall, a higher proportion of patients attained the NICE UK goal of ≤2.0 mmol/L (52.6%) than the more stringent ESC/EAS guideline goals of <1.4 mmol/L for very high-risk patients and <1.8 mmol/L for high-risk patients (32.9%). However, these observations run in parallel with the findings of low utilisation of combination therapies in UK patients in which at 1 year of follow-up, a higher proportion of patients had progressed on to LLT monotherapy from baseline (~10% increase, driven predominantly by an increased use of statins) than had progressed onto combination therapy (~2.2% increase). The observed low clinical utilisation of combination therapies appears inconsistent with published evidence that combination therapies improve LDL-C goal attainment, including other published data from the SANTORINI study but is consistent with UK NICE guidelines.^46 7 14^,^16^

While improvements in LDL-C were observed at 1-year follow-up in UK patients, the mean LDL-C measurements observed remained above the less stringent NICE UK goal of ≤2.0 mmol/L; in the group of other European countries, the mean LDL-C at 1-year follow-up was just below the NICE UK goal (1.98 mmol/L). The largest observed change in the UK dataset of LDL-C at 1 year relates to the addition of monotherapy to those patients on no LLT at baseline. As expected, this approach of introducing LLTs used as monotherapy reduces the LDL-C, but not sufficiently to get many patients to European or even NICE goals overall. This aligns with a parallel observation in the wider European dataset from the SANTORINI study.^7^ Compared with the European-wide data reported from the SANTORINI study, the proportions of LLTs received as monotherapies were much higher at baseline and at 1 year for UK patients than those observed across Europe (baseline–74.8% vs 53.6%, 1 year–84.9% vs 57.1%, respectively). Conversely, the observed use of LLTs as combination therapy was much lower for UK patients at baseline and at 1 year for UK patients than across Europe (baseline–4.9% vs 25.6%, 1 year–7.1% vs 37.9%, respectively).^7^ The observed proportion of patients on combination LLT was slightly higher than that recently observed in a population-level database study in Wales in which only 2.4% of n=16 387 patients received combination therapy in 2022 (population was specifically of patients with ASCVD).^17^ Of note, three-quarters of patients had no reported intensification of their LLT regimens during the 1-year follow-up, higher than the two-thirds of patients reported for the overall SANTORINI dataset.^7^ These findings seem to follow on from previous UK evidence asserting that up to 94% of patients with ASCVD and 85% of patients with high CV risk, without documented ASCVD, would require intensification of their treatment in order to achieve the 2014 NICE guidelines that recommended more intensive statin therapy.^18^ Given that the 2023 NICE guidelines were released after the SANTORINI study observation period, LDL-C attainment may have improved compared with that reported here. However, attainment would remain below the group of other European countries given that the NICE goals are not risk-status specific and are higher than the EAS/ESC LDL-C goals.

The relatively low uptake of combination LLT in the UK compared with the group of other European countries may also reflect several system-level influences. UK prescribing pathways and national guidance emphasise a stepwise approach in which statins are initiated first, with subsequent intensification occurring sequentially rather than through early adoption of combination regimens. This contrasts with the broader European context, where the 2019 ESC/EAS guidelines promoted earlier and more routine use of combination therapy to achieve lower LDL-C targets. In addition, differences in reimbursement processes, formulary access and prescriber familiarity with non-statin agents may contribute to slower uptake of combination LLT. These factors may help explain why combination therapy increased only modestly in the UK despite evidence supporting its efficacy and the larger increases observed elsewhere.

The modest treatment intensification during the 1 year of follow-up may reflect a lack of urgency to optimise LLT in asymptomatic patients. This also aligns with broader findings of low use of combination therapy and suggests that more proactive intensification may be required to improve LDL-C goal attainment in UK practice.

Since the beginning of the SANTORINI study, several additional non-statin LLTs have become available in the UK (eg, bempedoic acid and inclisiran), meaning that clinicians and patients have an increased array of treatment options available to them to help patients achieve their LDL-C goals.^19 20^ These treatment options, combined with UK guidelines and the National Guidance for Lipid Management, provide clinicians with multiple treatment pathways.^2 4 21^ The results of a recent UK-focused literature review, however, further confirm that despite the availability of several additional non-statin LLTs and relevant guidelines, a large proportion of patients being treated with LLT achieve suboptimal reductions in cholesterol, leaving them at high risk of further CV events.^22^,^27^ Overall, it is key to reduce LDL-C levels as low as possible to reduce CV risk, especially in patients with the highest levels of risk (evidence suggests that patients achieving LDL-C levels <1.8 mmol/L have reduced atherosclerotic plaque progression, compared with patients with LDL-C levels >1.8 mmol/L).^28^ These guideline goals are not ultimatums but relevant goals to provide both clinicians and patients with a common goal to work towards. Notably, the ESC/EAS have released their 2025 updated guidelines in which the guideline-recommended LDL-C goals remain the same, but there is a renewed focus on the utilisation of combination LLT therapy in order to bring down LDL-C levels.^29^

A recent UK study modelled a stepwise lipid management pathway (in line with the stepwise UK guidelines) that suggested that high-intensity statin monotherapy was not sufficient in most of their modelled scenarios to achieve guideline-recommended goals and provided suggestions for a novel treatment pathway in which the order that the drugs were added was important as to whether patients would reach goal LDL-C or not (eg, starting with monotherapy or combination therapy dependent on the patients’ baseline LDL-C levels and clinical/family history).^30^ Further research is needed to provide information as to the level of guideline LDL-C goal attainment in the years following the SANTORINI study, the update of guidelines and expansion of treatment options. Given the stepwise structure of the current treatment pathways, it is probable that the UK may continue to underutilise LLT combination therapies.

### Strengths and limitations

Aligned with the observational design of this study, several common biases are associated with this study, which were mitigated as best possible. A wide range of inclusion criteria was used to minimise selection bias, in addition to a large sample size and data monitoring processes to ensure the data was high quality and a high level of external validity. Although sites that participate in research can often be different from sites that do not participate. The SANTORINI study was not conducted specifically to assess attainment of the NICE 2023 LDL-C goal as this was introduced after the SANTORINI study had already been completed and thus the real-world attainment levels may have improved since this data was gathered. Due to the prospective nature of the study, the LDL-C attainment may have been impacted by the Hawthorne effect, though these results are strengthened by the sample size and the range of settings in which the data was gathered from. In line with the real-world aspect of the study, the analyses may have been limited by missing data, although this was mitigated as best possible during data collection. Of note, the lack of LDL-C data at follow-up reduced the LDL-C analysis population in this UK-focused SANTORINI study by ~40%. Although, this data was very similar to the overall population data regarding baseline characteristics and demographics. No formal statistical testing was conducted in this analysis and thus any inferences drawn from the descriptive analysis of groups should be treated with caution.

As lifestyle factors such as dietary modification, physical activity, weight change and smoking cessation can influence LDL-C levels, it is possible that unmeasured lifestyle changes contributed to some of the LDL-C variation observed over follow-up. Because lifestyle behaviours were not systematically captured in SANTORINI, their effect cannot be quantified within this analysis.

It is also important to acknowledge that the recruitment period (March 2020 to February 2021) coincided with the COVID-19 pandemic, during which routine healthcare delivery, appointment availability and lipid monitoring may have been disrupted. These circumstances may have influenced LLT prescribing decisions and follow-up testing patterns during this timeframe. Although the study design does not allow us to quantify the magnitude of this effect, it represents a potential contextual factor affecting real-world management.

## Conclusions

In this UK-focused analysis of data from the European SANTORINI study, we observed an increase in the use of LLT alongside a modest reduction in average LDL-C. However, mean LDL-C remained above the NICE UK goal threshold of ≤2 mmol/L at 1 year. The increase in LLT use was driven primarily by greater use of monotherapy, with only a modest rise in combination therapy utilisation. At 1-year follow-up, 53% of patients overall achieved the NICE UK goal compared with only 33% of patients who achieved the more stringent EAS/ESC risk-adjusted goals overall; the proportion is even lower in those patients with the highest unmet need (very high CV risk group) in which 56% achieved the NICE UK goal compared with 24% achieving the EAS/ESC risk-adjusted goal. Given the strong evidence supporting the efficacy of combination LLT in reducing LDL-C, this study suggests that there is an opportunity to improve the uptake of combination LLTs in the UK, which appeared to have a lower uptake relative to the other countries observed within the SANTORINI study. Improved uptake may subsequently improve the proportions of patients achieving guideline-recommended NICE and/or EAS/ESC LDL-C goals.

## Supplementary material

10.1136/bmjopen-2025-114031 online supplemental file 1

## Data Availability

Data are available on reasonable request.
