# Supplementary material for: Real-world changes in lipid-lowering therapy use and LDL-C goal attainment in high and very high cardiovascular risk patients in the UK: a secondary analysis of the European SANTORINI study 1-year follow-up
Source: BMJ Open. 2026 Apr 24;16(4):e114031. doi: 10.1136/bmjopen-2025-114031 (PMC13110627; doi:10.1136/bmjopen-2025-114031)
Supplement: online supplemental file 1 [file bmjopen-16-4-s001.docx]

Supplementary material for

**Real-world Changes in Lipid-Lowering Therapy Use and LDL-C Target Attainment in High and Very High Cardiovascular Risk Patients in the UK: A Secondary Analysis of the European SANTORINI Study 1-year follow-up**

Derek Connolly, Ahmet Fuat, Terry McCormack, Damien McNally, Jonathan Garstang, John Ryan, Amelia Reed, Daniel Robinson, Alberico L. Catapano, Kausik K. Ray

**Supplementary Figure 1.** Patient flow diagram

A patient could be included into both Primary Care and Secondary Care.

ASCVD, atherosclerotic cardiovascular disease; BAS, baseline analysis set; FAS, full analysis set; LDL-C, low-density lipoprotein cholesterol; LLT, lipid-lowering therapy

**Supplementary Figure 2.** Overall UK Lipid Lowering Therapy (LLT) at baseline and 1-year follow-up (%)

Key: LLT, lipid-lowering therapy; PCSK9i, proprotein convertase subtilisin/kexin type 9 inhibitor

**Combination therapy**

**Supplementary Table 1.** Baseline characteristics for overall, LDL-C patient population sets and by cardiovascular risk status

|  | **UK** | | | | **Other European countries** | | | |
| --- | --- | --- | --- | --- | --- | --- | --- | --- |
| **Characteristic** | **Overall**  **(N=634)** | **LDL-C dataset**  **(N=380)** | **High risk**  **(N= 346)** | **Very high risk**  **(N=287)** | **Overall**  **(N=8,502)** | **LDL-C dataset**  **(N=6,830)** | **High risk**  **(N= 2,280)** | **Very high risk**  **(N=6,217*** |
| **Male, n (%)** | 426 (67.2) | 247 (65.0) | 204 (59.0) | 221 (77.0) | 6,221 (73.2) | 4,950 (72.5) | 1,416 (62.1) | 4,802 (77.2) |
| **Age, years, mean (SD)** | 67.0 (9.0) | 66.8 (8.6) | 65.8 (8.9) | 68.5 (8.9) | 65.3 (11.0) | 64.9 (11.0) | 63.2 (12.1) | 66.1 (10.4) |
| **Risk classification assigned by investigator, n (%)**  Missing risk  Very high risk  High risk | 1 (0.2)  287 (45.3)  346 (54.6) | 0 (0.0)  181 (47.6)  199 (52.4) | - | - | 5 (0.1)  6,217 (73.1)  2,280 (26.8) | 4 (0.1)  4,992 (73.1)  1,834 (26.9) | - | - |
| **ASCVD, n (%)** | 275 (43.4) | 167 (44.0) | 106 (30.6) | 168 (58.6) | 6,794 (79.9) | 5,354 (78.4) | 986 (43.3) | 5,805 (93.4) |
| **BMI, mean (SD)** | 30.3 (6.1) | 29.98 (5.7) | 30.61 (6.3) | 29.80 (5.7) | 28.20 (4.8) | 28.14 (4.8) | 28.21 (5.0) | 28.20 (4.8) |
| **Systolic blood pressure, mmHg, mean (SD)** | 133.7 (15.9) | 133.1 (15.7) | 134.2 (16.0) | 133.2 (15.8) | 134.1 (18.2) | 133.8 (17.9) | 134.7 (17.9) | 133.8 (18.4) |
| **Diastolic blood pressure, mmHg, mean (SD)** | 76.6 (10.2) | 76.4 (10.1) | 77.5 (10.1) | 75.7 (10.3) | 78.0 (10.5) | 78.1 (10.3) | 78.9 (10.2) | 77.7 (10.6) |
| **Hypertension, n (%)** | 404 (63.7) | 225 (59.2) | 223 (64.5) | 180 (62.7) | 6,104 (71.8) | 4,865 (71.2) | 1,522 (66.8) | 4,580 (73.7) |
| **Diabetes, n (%)** | 272 (42.9) | 162 (42.6) | 150 (43.4) | 121 (42.2) | 2,920 (34.3) | 2,353 (34.5) | 764 (33.5) | 2,155 (34.7) |
| **eGFR, mL/min/1.73m^2^, mean (SD)** | 76.0 (22.6) | 76.7 (21.2) | 79.7 (22.8) | 71.9 (21.6) | 78.1 (24.2) | 78.9 (23.9) | 81.7 (24.4) | 76.89 (24.0) |
| **Heterozygous familial hypercholesterolemia, n (%)** | 41 (6.5) | 25 (6.6) | 18 (5.2) | 23 (8.0) | 893 (10.5) | 775 (11.4) | 413 (18.1) | 480 (7.7) |
| **All Smoking history, n (%)**  Current  Former  Never | 86 (13.6)  298 (47.0)  249 (39.3) | 48 (12.6)  174 (45.8)  157 (41.3) | 49 (14.2)  163 (47.1)  133 (38.4) | 37 (12.9)  134 (46.7)  116 (40.4) | 1,418 (16.7)  3,580 (42.1)  3,415 (40.2) | 1,114 (16.3)  2,858 (41.9)  2,800 (41.0) | 346 (15.2)  762 (33.4)  1,153 (50.6) | 1,071 (17.2)  2,816 (45.3)  2,261 (36.4) |
| **LDL-C, mean (SD)**  mmol/L  mg/dL | 2.4 (1.2)  94.2 (46.2) | 2.5 (1.2)  96.9 (47.1) | 2.6 (1.2)  98.5 (47.5) | 2.3 (1.1)  89.5 (44.2) | 2.4 (1.2)  92.7 (46.6) | 2.4 (1.2)  93.3 (47.1) | 2.7 (1.3)  104.2 (50.5) | 2.3 (1.1)  88.5 (44.2) |
| **Site characteristics, n (%)**  Primary care  Secondary care | 535 (84.4)  99 (15.6) | - | 294 (85.0)  52 (15.0) | 241 (84.0)  46 (16.0) | 2,740 (32.2)  6,927 (81.5) | 2,286 (33.5)  5,442 (79.7) | 1,080 (47.4)  1,606 (70.4) | 1,660 (26.7)  5,316 (85.5) |

Key: ASCVD, atherosclerotic cardiovascular disease; BMI, body mass index; eGFR, estimated glomerular filtration rate; LDL-C, low-density lipoprotein cholesterol; SD, standard deviation. Note. Not all patients had risk data available, and thus the number of patients in the risk-stratified groups is fewer than that in the overall cohort.

**Supplementary Table 2.** LLTs at baseline and 1-year follow-up in overall, patients with high CV risk and very high CV risk (UK Full Analysis Set)

|  | **Overall (N=634)** | | **High CV risk (N=346)** | | **Very high CV risk (N=287)** | |
| --- | --- | --- | --- | --- | --- | --- |
| **LLT, n (%)** | **Baseline** | **1-year follow-up** | **Baseline** | **1-year follow-up** | **Baseline** | **1-year follow-up** |
| **Missing** | 0 (0.00) | 6 (0.95) | 0 (0.00) | 2 (0.58) | 0 (0.00) | 4 (1.39) |
| **No LLT** | 129 (20.35) | 45 (7.10) | 81 (23.41) | 33 (9.54) | 48 (16.72) | 12 (4.18) |
| **Total monotherapy** | 474 (74.76) | 538 (84.86) | 257 (74.28) | 293 (84.69) | 216 (75.26) | 244 (85.02) |
| **Statin alone**  Missing intensity  Low intensity  Moderate intensity  High intensity | 448 (70.66)  4 (0.63)  7 (1.10)  278 (43.85)  159 (25.08) | 513 (80.91)  2 (0.32)  4 (0.63)  311 (49.05)  196 (30.91) | 246 (71.10)  3 (0.87)  2 (0.58)  172 (49.71)  69 (19.94) | 282 (81.50)  1 (0.29)  1 (0.29)  194 (56.07)  86 (24.86) | 201 (70.03)  1 (0.35)  5 (1.74)  105 (36.59)  90 (31.36) | 230 (80.14)  1 (0.35)  3 (1.05)  116 (40.42)  110 (38.33) |
| **Ezetimibe alone** | 15 (2.37) | 10 (1.58) | 7 (2.02) | 4 (1.16) | 8 (2.79) | 6 (2.09) |
| **PCSK9i alone** | 7 (1.10) | 12 (1.89) | 2 (0.58) | 5 (1.45) | 5 (1.74) | 7 (2.44) |
| **Bempedoic acid alone** | 0 (0.00) | 1 (0.16) | 0 (0.00) | 1 (0.29) | 0 (0.00) | 0 (0.00) |
| **Any other oral LLT alone** | 4 (0.63) | 2 (0.32) | 2 (0.58) | 1 (0.29) | 2 (0.70) | 1 (0.35) |
| **Total combination therapy** | 31 (4.89) | 45 (7.10) | 8 (2.32) | 18 (5.20) | 23 (8.01) | 27 (9.41) |
| **Combination statin + ezetimibe**  Missing intensity statin  Low intensity statin  Moderate intensity statin  High intensity statin | 16 (2.52)  1 (0.16)  1 (0.16)  3 (0.47)  11 (1.74) | 22 (3.47)  0 (0.00)  3 (0.47)  5 (0.79)  14 (2.21) | 4 (1.16)  1 (0.29)  0 (0.00)  0 (0.00)  3 (0.87) | 8 (2.31)  0 (0.00)  1 (0.29)  1 (0.29)  6 (1.73) | 12 (4.18)  0 (0.00)  1 (0.35)  3 (1.05)  8 (2.79) | 14 (4.88)  0 (0.00)  2 (0.70)  4 (1.39)  8 (2.79) |
| **BA combination** | 0 (0.00) | 1 (0.16) | 0 (0.00) | 1 (0.29) | 0 (0.00) | 0 (0.00) |
| **PCSK9i combination** | 9 (1.42) | 15 (2.37) | 3 (0.87) | 7 (2.02) | 6 (2.09) | 8 (2.79) |
| **Any other combination therapy** | 6 (0.95) | 7 (1.10) | 1 (0.29) | 2 (0.58) | 5 (1.74) | 5 (1.74) |

Key: BA, bempedoic acid; FDC, fixed dose combination; LLT, lipid-lowering therapy; PCSK9i, proprotein convertase subtilisin/kexin type 9 inhibitor

**Supplementary Table 3.** LLTs at baseline and 1-year follow-up in overall, patients with high CV risk and very high CV risk across a group of other European countries* (Full Analysis Set)

|  | **Overall (N=8502)** | | | **Very high risk (N=6217)** | | **High risk (N=2280)** | |
| --- | --- | --- | --- | --- | --- | --- | --- |
| **LLT, n (%)** | **Baseline n (%)** | | **1-year follow-up n (%)** | **Baseline n (%)** | **1-year follow-up n (%)** | **Baseline n (%)** | **1-year follow-up n (%)** |
| **Missing** | 0 (0.00) | | 149 (1.75) | 0 (0.00) | 119 (1.91) | 0 (0.00) | 30 (1.32) |
| **No LLT documented** | 1780 (20.94) | | 258 (3.03) | 1259 (20.25) | 138 (2.22) | 517 (22.68) | 119 (5.22) |
| **Total monotherapy** | 4418 (51.96) | | 4676 (55.00) | 3147 (50.62) | 3230 (51.95) | 1270 (55.70) | 1442 (63.25) |
| **Statin alone** | 4068 (47.85) | | 4299 (50.56) | 2896 (46.58) | 2963 (47.66) | 1171 (51.36) | 1332 (58.42) |
| Missing intensity | 85 (1.00) | | 78 (0.92) | 54 (0.87) | 53 (0.85) | 31 (1.36) | 25 (1.10) |
| Low intensity | 128 (1.51) | | 112 (1.32) | 82 (1.32) | 66 (1.06) | 46 (2.02) | 46 (2.02) |
| Moderate intensity | 2053 (24.15) | | 1947 (22.90) | 1329 (21.38) | 1154 (18.56) | 724 (31.75) | 790 (34.65) |
| High intensity | 1802 (21.20) | | 2162 (25.43) | 1431 (23.02) | 1690 (27.18) | 370 (16.23) | 471 (20.66) |
| **Ezetimibe alone** | 155 (1.82) | | 136 (1.60) | 109 (1.75) | 87 (1.40) | 46 (2.02) | 49 (2.15) |
| **Bempedoic acid alone** | 0 (0.00) | | 10 (0.12) | 0 (0.00) | 10 (0.16) | 0 (0.00) | 0 (0.00) |
| **PCSK9i alone** | 144 (1.69) | | 190 (2.23) | 114 (1.83) | 150 (2.41) | 30 (1.32) | 40 (1.75) |
| **Any other oral LLT alone** | 51 (0.60) | | 41 (0.48) | 28 (0.45) | 20 (0.32) | 23 (1.01) | 21 (0.92) |
| **Total combination therapy** | 2304 (27.10) | | 3419 (40.21) | 1811 (29.13) | 2730 (43.91) | 493 (21.62) | 689 (30.22) |
| **Combination statin + ezetimibe** | 1545 (18.17) | | 2392 (28.13) | 1232 (19.82) | 1955 (31.45) | 313 (13.73) | 437 (19.17) |
| Missing intensity | 42 (0.49) | | 56 (0.66) | 35 (0.56) | 44 (0.71) | 7 (0.31) | 12 (0.53) |
| Low intensity | 36 (0.42) | | 36 (0.42) | 28 (0.45) | 28 (0.45) | 8 (0.35) | 8 (0.35) |
| Moderate intensity | 541 (6.36) | | 701 (8.25) | 414 (6.66) | 528 (8.49) | 127 (5.57) | 173 (7.59) |
| High intensity | 926 (10.89) | | 1599 (18.81) | 755 (12.14) | 1355 (21.80) | 171 (7.50) | 244 (10.70) |
| **Bempedoic acid combination therapy** | 0 (0.00) | | 18 (0.21) | 0 (0.00) | 15 (0.24) | 0 (0.00) | 3 (0.13) |
| **Bempedoic acid FDC alone** | 1 (0.01) | | 15 (0.18) | 0 (0.00) | 9 (0.14) | 1 (0.04) | 6 (0.26) |
| **Bempedoic acid FDC combination with statin** | 1 (0.01) | | 19 (0.22) | 0 (0.00) | 15 (0.24) | 1 (0.04) | 4 (0.18) |
| **PCSK9i combination** | 421 (4.95) | | 585 (6.88) | 325 (5.23) | 450 (7.24) | 96 (4.21) | 135 (5.92) |
| **Any other oral combination LLT** | 336 (3.95) | | 390 (4.59) | 254 (4.09) | 286 (4.60) | 82 (3.60) | 104 (4.56) |
|  | |  |  |  |  |  |  |

**Supplementary Table 4.** Treatment intensification classification (FAS)

|  | **UK** | | | **Other European Countries*** | | |
| --- | --- | --- | --- | --- | --- | --- |
| **Classification** | **Overall (N=634)** | **High CV risk (N=346)** | **Very High CV risk (N=287)** | **Overall**  **(N=8,502)** | **High CV risk**  **(N=2,280)** | **Very high CV risk**  **(N=6,217)** |
| Missing, n (%) | 6 (0.95) | 2 (0.58) | 4 (1.39) | 149 (1.75) | 30 (1.32) | 119 (1.91) |
| No change, n (%) | 478 (75.39) | 252 (72.83) | 225 (78.4) | 5,602 (65.89) | 1,602 (70.26) | 3,999 (64.32) |
| De-Escalation, n (%) | 16 (2.52) | 13 (3.76) | 3 (1.05) | 211 (2.48) | 44 (1.93) | 166 (2.67) |
| Escalation, n (%) | 134 (21.14) | 79 (22.83) | 55 (19.16) | 2,540 (29.88) | 604 (26.49) | 1,933 (31.09) |

Key: CV, cardiovascular

*Includes the other European countries included in the SANTORINI study.
