# Supplementary material for: Real-world changes in lipid-lowering therapy use and LDL-C goal attainment in high and very high cardiovascular risk patients in the UK: a secondary analysis of the European SANTORINI study 1-year follow-up
Source: BMJ Open. 2026 Apr 24;16(4):e114031. doi: 10.1136/bmjopen-2025-114031 (PMC13110627; doi:10.1136/bmjopen-2025-114031)
Supplement: Supplementary file 2 [file bmjopen-2025-114031.reviewer_comments.pdf]

PEER REVIEW HISTORY

BMJ Open publishes all reviews undertaken for accepted manuscripts. Reviewers are asked to complete a checklist review form and are provided with free text boxes to elaborate on their assessment. These free text comments are reproduced below.

ARTICLE DETAILS

Title (Provisional)

Real-world Changes in Lipid-Lowering Therapy Use and LDL-C Goal Attainment in High and Very High Cardiovascular Risk Patients in the UK: A Secondary Analysis of the European SANTORINI Study 1-year follow-up

Authors

Connolly, Derek; Fuat, Ahmet; McCormack, Terry; Mcnally, Damien; Garstang, Jonathan; Ryan, John; Reed, Amelia; Robinson, Daniel; Catapano, Alberico; Ray, Kausik K

VERSION 1 - REVIEW

Reviewer 1

Name Yang, Yuehang

Affiliation Huazhong University of Science and Technology Tongji Medical College Tongji Hospital, Cardiovascular Surgery

Date 13-Jan-2026

COI None

This is a well-conducted and clearly reported UK-focused secondary analysis of a large European real-world cohort, providing clinically and policy-relevant insights into lipid-lowering therapy use. The following minor comments are intended to further strengthen the manuscript.

1.The Conclusion section of the abstract appears relatively long and partly overlaps with the Results. Consider streamlining the conclusion to focus on the key descriptive findings.

2.The comparisons between the UK and other European countries are informative. The authors may consider adding brief discussion on possible factors that could be associated with the differing 1-year changes in combination lipid-lowering therapy use observed between the UK (4.89% to 7.10%) and other European countries (27.10% to 40.21%). Consideration of healthcare system characteristics, reimbursement policies, or prescribing practices may help place these findings in a broader context.

3.The authors may consider including a brief statement in the Methods section indicating adherence to the STROBE reporting guidelines, which may help to enhance the clarity and transparency of reporting.

**Reviewer**	**2**
**Name**	**Mashili, Fredirick**
**Affiliation**	**Muhimbili University of Health and Allied Sciences, Physiology**
**Date**	**21-Feb-2026**
**COI**	**None**

The authors aimed to evaluate changes in lipid-lowering therapy (LLT) utilisation and LDL-C goal attainment over one year among UK patients with high or very high cardiovascular (CV) risk, using data from the prospective, observational study. The analysis further analysed UK prescribing patterns by comparison with other participating European countries.

Using prospectively collected real-world data from 663 UK patients (with LDL-C data available in 380 patients at both baseline and 1-year follow-up), the authors describe LLT use (monotherapy vs combination therapy), treatment intensification patterns, and attainment of NICE and ESC/EAS LDL-C targets.

Over one year, UK patients showed reduced non-use of LLT, increased monotherapy and modestly increased combination therapy use, leading to a modest reduction in LDL-C with combination therapy uptake remaining lower than in other European countries.

The manuscript is clearly written, logically structured, and based on an impressively large prospective dataset reflecting real-world clinical practice.

The study’s key strengths include its large prospective real-world multinational dataset, clear reporting of prescribing patterns and LDL-C goal attainment stratified by risk, and a transparent, appropriately descriptive analytical approach.

Major Comments

1. Consideration of Lifestyle Factors

While the manuscript appropriately focuses on pharmacologic LLT patterns, it does not acknowledge the potential contribution of lifestyle factors to changes in LDL-C observed over follow-up.

From the Methods section, data collected included medical history, LLT use, co-medications, LDL-C values, and treatment escalation/de-escalation. However, there appears to be no collection or analysis of:

Dietary modification

Physical activity

Weight change over follow-up

Smoking or alcohol behaviour changes

Participation in cardiac rehabilitation or lifestyle counselling

Given that LDL-C levels improved modestly over one year in this observational setting, it would be important to acknowledge that these changes cannot be attributed solely to pharmacologic treatment modification.

I recommend adding a statement in the limitations section clarifying whether lifestyle variables were not captured and acknowledging their potential influence on LDL-C outcomes. This would strengthen the interpretative completeness of the manuscript without altering its conclusions.

Minor Comments

1. Clarification of Monotherapy Increase

The manuscript reports an increase in LLT monotherapy use from baseline to 1 year in the UK cohort. It would enhance interpretability to clarify whether this increase primarily reflects:

Initiation of therapy among previously untreated patients,

Escalation from lower-intensity therapy,

De-escalation from combination therapy, or

Switching between drug classes.

Based on the presented data, the reduction in “no LLT” appears to account for most of the monotherapy increase, but a brief clarification in the text (or a transition table summarising baseline-to-follow-up category shifts) would make this explicit.

2. Treatment Intensification Reporting

Supplementary Table 3 presents valuable data on treatment escalation, de-escalation, and no change in treatment intensity. These findings are important for understanding the mechanisms underlying LDL-C changes but are somewhat under-emphasised in the main Results narrative.

Consider highlighting these intensification patterns more explicitly and linking them directly to observed LDL-C changes.

3. Interpretation of Low Combination Therapy Uptake in the UK

The manuscript appropriately notes the lower uptake of combination LLT in the UK compared with other European countries. It may strengthen the Discussion to briefly

consider potential system-level explanations, such as prescribing culture, reimbursement policies, access considerations, or guideline implementation pathways.

This would add contextual depth without over-speculating.

Given that recruitment occurred between March 2020 and February 2021, the study period overlaps substantially with the COVID-19 pandemic. A brief acknowledgement of possible healthcare system disruption or altered clinical follow-up during this period may be warranted.

VERSION 1 - AUTHOR RESPONSE

Reviewer: 1
Dr. Yuehang Yang, Huazhong University of Science and Technology Tongji Medical College Tongji Hospital, Huazhong University of Science and Technology Tongji Medical College Union Hospital

Comments to the Author:
This is a well-conducted and clearly reported UK-focused secondary analysis of a large European real-world cohort, providing clinically and policy-relevant insights into lipid-lowering therapy use. The following minor comments are intended to further strengthen the manuscript.

1. The Conclusion section of the abstract appears relatively long and partly overlaps with the Results. Consider streamlining the conclusion to focus on the key descriptive findings.

Authors' response: Thank you for this helpful observation. We have revised it to provide a more concise, high-level summary focused on the key descriptive findings without repeating detailed numerical results.

> "*Conclusions*: In this UK-focused analysis of the SANTORINI study, use of lipid-lowering therapy increased modestly over one year, accompanied by a reduction in average LDL-C levels. However, mean LDL-C remained above the NICE goal, and attainment of both NICE and ESC/EAS LDL-C thresholds remained suboptimal. The findings highlight continued opportunities to optimise lipid management in the UK clinical practice, including the potential for broader use of combination therapies."

2. The comparisons between the UK and other European countries are informative. The authors may consider adding brief discussion on possible factors that could be associated with the differing 1-year changes in combination lipid-lowering therapy use observed between the UK (4.89% to 7.10%) and other European countries (27.10% to 40.21%). Consideration of healthcare system characteristics, reimbursement policies, or prescribing practices may help place these findings in a broader context.

Authors' response: Thank you for your helpful comment which was mentioned by the other reviewer as well. We agree that contextualising the low uptake of combination LLT in the UK would strengthen the Discussion. These include the structure of current UK prescribing pathways (which prioritise stepwise escalation beginning with statin

monotherapy), differences in guideline implementation timelines relative to the 2019 ESC/EAS recommendations, and practical access and reimbursement considerations that influence the adoption of non-statin LLTs. We have therefore added a paragraph outlining system-level factors that may help explain this pattern:

> "The relatively low uptake of combination LLT in the UK compared with the group of other European countries may also reflect several system-level influences. UK prescribing pathways and national guidance emphasise a stepwise approach in which statins are initiated first, with subsequent intensification occurring sequentially rather than through early adoption of combination regimens. This contrasts with the broader European context, where the 2019 ESC/EAS guidelines promoted earlier and more routine use of combination therapy to achieve lower LDL-C targets. In addition, differences in reimbursement processes, formulary access, and prescriber familiarity with non-statin agents may contribute to slower uptake of combination LLT. These factors may help explain why combination therapy increased only modestly in the UK despite evidence supporting its efficacy and the larger increases observed elsewhere."

3.The authors may consider including a brief statement in the Methods section indicating adherence to the STROBE reporting guidelines, which may help to enhance the clarity and transparency of reporting.

Authors' response: Thank you for this suggestion. We have added a brief statement to the Methods section indicating that the study reporting follows the STROBE recommendations for observational research.

> "Reporting of this observational study adheres to the STROBE (Strengthening the Reporting of Observational Studies in Epidemiology) guidelines."

Reviewer: 2
Dr. Fredirick Mashili, Muhimbili University of Health and Allied Sciences

Comments to the Author:
The authors aimed to evaluate changes in lipid-lowering therapy (LLT) utilisation and LDL-C goal attainment over one year among UK patients with high or very high cardiovascular (CV) risk, using data from the prospective, observational study. The analysis further analysed UK prescribing patterns by comparison with other participating European countries.
Using prospectively collected real-world data from 663 UK patients (with LDL-C data available in 380 patients at both baseline and 1-year follow-up), the authors describe LLT use (monotherapy vs combination therapy), treatment intensification patterns, and attainment of NICE and ESC/EAS LDL-C targets.

Over one year, UK patients showed reduced non-use of LLT, increased monotherapy and modestly increased combination therapy use, leading to a modest reduction in LDL-C with combination therapy uptake remaining lower than in other European countries.
The manuscript is clearly written, logically structured, and based on an impressively large prospective dataset reflecting real-world clinical practice.

The study's key strengths include its large prospective real-world multinational dataset, clear reporting of prescribing patterns and LDL-C goal attainment stratified by risk, and a transparent, appropriately descriptive analytical approach.

Major Comments

1. Consideration of Lifestyle Factors

While the manuscript appropriately focuses on pharmacologic LLT patterns, it does not acknowledge the potential contribution of lifestyle factors to changes in LDL-C observed over follow-up.

Authors' response: Thank you for this suggestion. We agree that lifestyle factors such as diet, physical activity, weight change, and smoking status may also influence LDL-C levels over time. As the SANTORINI study did not systematically capture lifestyle changes during follow-up, we are unable to quantify their contribution to the observed LDL-C reductions. However, we have added an acknowledgement in the Discussion to note that lifestyle modification represents a potential unmeasured influence on LDL-C trajectories in this real-world cohort:

> "As lifestyle factors such as dietary modification, physical activity, weight change, and smoking cessation can influence LDL-C levels, it is possible that unmeasured lifestyle changes contributed to some of the LDL-C variation observed over follow-up. Because lifestyle behaviours were not systematically captured in SANTORINI, their effect cannot be quantified within this analysis."

From the Methods section, data collected included medical history, LLT use, co-medications, LDL-C values, and treatment escalation/de-escalation. However, there appears to be no collection or analysis of:

Dietary modification

Physical activity

Weight change over follow-up

Smoking or alcohol behaviour changes

Participation in cardiac rehabilitation or lifestyle counselling

Given that LDL-C levels improved modestly over one year in this observational setting, it would be important to acknowledge that these changes cannot be attributed solely to pharmacologic treatment modification.

Authors' response: Thank you for highlighting this point. We agree that lifestyle factors may influence LDL-C levels over time. As noted, the SANTORINI study did not systematically collect these variables during follow-up, and therefore the present analysis cannot assess their contribution to the modest LDL-C improvements observed. We have added a statement in the 'Strengths and Limitations' section acknowledging that LDL-C changes in this observational context cannot be attributed solely to pharmacologic treatment modification, given the absence of lifestyle data. This enhances the interpretive clarity of the manuscript without altering its conclusions.

> "As lifestyle factors such as dietary modification, physical activity, weight change, and smoking cessation can influence LDL-C levels, it is possible that unmeasured lifestyle changes contributed to some of the LDL-C variation observed over follow-up. Because lifestyle behaviours were not systematically captured in SANTORINI, their effect cannot be quantified within this analysis."

and

"The modest treatment intensification during the 1 year of follow-up may reflect a lack of urgency to optimize LLT in asymptomatic patients. This also aligns with broader findings of low use of combination therapy and suggests that more proactive intensification may be required to improve LDL-C goal attainment in UK practice."

I recommend adding a statement in the limitations section clarifying whether lifestyle variables were not captured and acknowledging their potential influence on LDL-C outcomes. This would strengthen the interpretative completeness of the manuscript without altering its conclusions.

Authors' response: Thank you for this helpful recommendation. As noted in our response to the previous comment, we have now added a statement to the Strengths and Limitations section acknowledging that lifestyle variables (e.g., diet, physical activity, weight change, smoking) were not systematically captured in the SANTORINI study and may have contributed to LDL-C changes.

Minor Comments

1. Clarification of Monotherapy Increase

The manuscript reports an increase in LLT monotherapy use from baseline to 1 year in the UK cohort. It would enhance interpretability to clarify whether this increase primarily reflects:

Initiation of therapy among previously untreated patients,
Escalation from lower-intensity therapy,
De-escalation from combination therapy, or
Switching between drug classes.

Based on the presented data, the reduction in "no LLT" appears to account for most of the monotherapy increase, but a brief clarification in the text (or a transition table summarising baseline-to-follow-up category shifts) would make this explicit.

Authors' response: Thank you for your helpful comment. We have clarified the drivers of the observed increase in monotherapy in the UK cohort using the presented category counts. Between baseline and 1 year (UK FAS, n = 634), 'no LLT' decreased from 129 (20.4%) to 45 (7.1%), monotherapy increased from 474 (74.8%) to 538 (84.9%), and combination therapy increased from 31 (4.9%) to 45 (7.1%). This net pattern indicates that initiation among previously untreated patients accounted for most of the observed rise in monotherapy, with a smaller contribution from category changes. Consistent with the treatment-intensification summary (Supplementary Table 3), de-escalation events were infrequent (16/634; 2.5%), and escalation occurred largely within monotherapy intensity (which does not change the monotherapy category; the number of escalations (134) is far larger than the increase in combination therapy (+14)).

We have added clarifying text in the Results:

"In the UK FAS (n = 634), category counts moved from baseline to 1-year as follows: 'no LLT' 129 (20.4%) to 45 (7.1%), monotherapy 474 (74.8%) to 538 (84.9%), and combination therapy 31 (4.9%) to 45 (7.1%); 6 participants were missing at follow-up (Supplementary Table 1). This net pattern indicates that the

increase in monotherapy was driven predominantly by initiation among previously untreated patients, with only a minor contribution from category changes. In keeping with the treatment-intensification summary (75.4% no change; 21.1% escalation; 2.5% de-escalation), most escalation occurred within monotherapy intensity brackets (Supplementary Table 3) and therefore did not alter treatment category, while de-escalation from combination to monotherapy was uncommon."

2. Treatment Intensification Reporting
Supplementary Table 3 presents valuable data on treatment escalation, de-escalation, and no change in treatment intensity. These findings are important for understanding the mechanisms underlying LDL-C changes but are somewhat under-emphasised in the main Results narrative.
Consider highlighting these intensification patterns more explicitly and linking them directly to observed LDL-C changes.

Authors' response: Thank you for this helpful suggestion. We agree that the treatment-intensification findings provide important context for understanding the observed LDL-C changes. We have therefore expanded the Results section to highlight these treatment-intensification patterns more explicitly and now refer directly to the relevant supplementary tables within the text. Due to journal table/figure limitations, and to maintain clarity in the main manuscript, we have not duplicated the intensification table in the main body but have retained it in the Supplementary Materials.

"Patterns of treatment intensification also contribute to understanding the LDL-C changes observed at 1 year. As shown in Supplementary Table 3, most UK patients had no change in their treatment intensity (478/634; 75.4%), while 134 (21.1%) experienced escalation and 16 (2.5%) underwent de-escalation. These escalation events predominantly increased the intensity of existing monotherapy regimens, as reflected by the small rise in combination therapy use (+14 patients). When considered alongside the substantial reduction in patients on no LLT (129 to 45), these findings indicate that the LDL-C reductions observed in the UK cohort were driven mainly by initiation of therapy in previously untreated individuals and intensification within monotherapy, rather than by widespread adoption of combination therapy."

3. Interpretation of Low Combination Therapy Uptake in the UK
The manuscript appropriately notes the lower uptake of combination LLT in the UK compared with other European countries. It may strengthen the Discussion to briefly consider potential system-level explanations, such as prescribing culture, reimbursement policies, access considerations, or guideline implementation pathways.
This would add contextual depth without over-speculating.

Authors' response: Thank you for this constructive suggestion, which was mentioned by the other reviewer as well. We agree that contextualising the low uptake of combination LLT in the UK would strengthen the Discussion. These include the structure of current UK prescribing pathways (which prioritise stepwise escalation beginning with statin monotherapy), differences in guideline implementation timelines relative to the 2019 ESC/EAS recommendations, and practical access and reimbursement considerations that influence the adoption of non-statin LLTs. We have therefore added a paragraph outlining system-level factors that may help explain this pattern:

"The relatively low uptake of combination LLT in the UK compared with the group of other European countries may also reflect several system-level influences. UK prescribing pathways and national guidance emphasise a stepwise approach in which statins are initiated first, with subsequent intensification occurring sequentially rather than through early adoption of combination regimens. This contrasts with the broader European context, where the 2019 ESC/EAS guidelines promoted earlier and more routine use of combination therapy to achieve lower LDL-C targets. In addition, differences in reimbursement processes, formulary access, and prescriber familiarity with non-statin agents may contribute to slower uptake of combination LLT. These factors may help explain why combination therapy increased only modestly in the UK despite evidence supporting its efficacy and the larger increases observed elsewhere."

Given that recruitment occurred between March 2020 and February 2021, the study period overlaps substantially with the COVID-19 pandemic. A brief acknowledgement of possible healthcare system disruption or altered clinical follow-up during this period may be warranted.

Authors' response: Thank you for this important point. We agree that the overlap between the recruitment period (March 2020–February 2021) and the COVID-19 pandemic may have influenced clinical practice and follow-up. We have added a brief statement acknowledging this to Strengths and Limitations section.

"It is also important to acknowledge that the recruitment period (March 2020 to February 2021) coincided with the COVID-19 pandemic, during which routine healthcare delivery, appointment availability, and lipid monitoring may have been disrupted. These circumstances may have influenced LLT prescribing decisions and follow-up testing patterns during this timeframe. Although the study design does not allow us to quantify the magnitude of this effect, it represents a potential contextual factor affecting real-world management."

Reviewer: 1
If you have selected 'Yes' above, please provide details of any competing interests.: Not applicable

Reviewer: 2
If you have selected 'Yes' above, please provide details of any competing interests.: Notapplicable

Commented [OB1]: ''This aligns with our broader finding of low use of combi therapy and suggests that more proactive intensification may be required to improve LDL-C goal attainment in UK practice'' Maybe something linking these two ideas
